# Novel peripheral blood diagnostic biomarkers screened by machine learning algorithms in ankylosing spondylitis

**DOI:** 10.3389/fgene.2022.1032010

**Published:** 2022-11-01

**Authors:** Jian Wen, Lijia Wan, Xieping Dong

**Affiliations:** ^1^ Medical College of Nanchang University, Nanchang, Jiangxi, China; ^2^ JXHC Key Laboratory of Digital Orthopedics, Department of Orthopedics, Jiangxi Provincial People’s Hospital, The First Affiliated Hospital of Nanchang Medical College, Nanchang, Jiangxi, China; ^3^ Department of Child Healthcare, Hunan Provincial Maternal and Child Health Hospital, Changsha, Hunan, China

**Keywords:** machine learning, ankylosing spondylitis, diagnostic model, immune microenvironment, informatics

## Abstract

**Background:** Ankylosing spondylitis (AS) is a chronic inflammatory disorder of unknown etiology that is hard to diagnose early. Therefore, it is imperative to explore novel biomarkers that may contribute to the easy and early diagnosis of AS.

**Methods:** Common differentially expressed genes between normal people and AS patients in GSE73754 and GSE25101 were screened by machine learning algorithms. A diagnostic model was established by the hub genes that were screened. Then, the model was validated in several data sets.

**Results:** *IL2RB* and *ZDHHC18* were screened using machine learning algorithms and established as a diagnostic model. Nomograms suggested that the higher the expression of *ZDHHC18*, the higher was the risk of AS, while the reverse was true for *IL2RB in vivo*. C-indexes of the model were no less than 0.84 in the validation sets. Calibration analyses suggested high prediction accuracy of the model in training and validation cohorts. The area under the curve (AUC) values of the model in GSE73754, GSE25101, GSE18781, and GSE11886 were 0.86, 0.84, 0.85, and 0.89, respectively. The decision curve analyses suggested a high net benefit offered by the model. Functional analyses of the differentially expressed genes indicated that they were mainly clustered in immune response–related processes. Immune microenvironment analyses revealed that the neutrophils were expanded and activated in AS while some T cells were decreased.

**Conclusion:** *IL2RB* and *ZDHHC18* are potential blood biomarkers of AS, which might be used for the early diagnosis of AS and serve as a supplement to the existing diagnostic methods. Our study deepens the insight into the pathogenesis of AS.

## Introduction

Ankylosing spondylitis (AS), also known as radiographic axial spondyloarthritis, is one of the two types of axial spondyloarthritides (Sieper et al., 2015; Taurog et al., 2016; Sieper and Poddubnyy, 2017; Navarro-Compán et al., 2021). It is a chronic inflammatory disorder mainly affecting the axial joints and entheses and is usually characterized by typical features such as inflammatory back pain, limitation of the motion of the lumbar spine, restricted chest expansion, and advanced sacroiliitis on plain radiographs. Some patients with AS also experience peripheral spondyloarthritis symptoms such as dactylitis and Achilles tendinitis and extra-articular manifestations such as uveitis, psoriasis, inflammatory bowel disease, and many others, either simultaneously or at some point during the course of the disease. The diagnosis of AS is based on the Modified New York criteria: advanced sacroiliitis on plain radiographs with any one of the three typical aforementioned features (van der Linden et al., 1984). Patients usually do not meet the criterion of advanced sacroiliitis on plain radiographs; however, those with sacroiliitis on MRI or HLA-B27 positivity plus the clinical criteria are classified into non-radiographic axial spondyloarthritis (Rudwaleit et al., 2009; Rudwaleit et al., 2011).

The prevalence of AS, which reportedly varies with geography, ranges from 0.02–0.35%, while that of axial spondyloarthritis is estimated to be 0.20–1.61%, which is much higher than the prevalence of AS, indicating a high ratio of non-radiographic axial spondyloarthritis patients (Dean et al., 2014; Stolwijk et al., 2016; Ward et al., 2019). Especially, with the development of diagnostic tools and further understanding of axial spondyloarthritis, patients without advanced sacroiliitis on plain radiographs raise more attention, and more non-radiographic axial spondyloarthritides are detected together with updates in its definition (Taurog et al., 2016; Ritchlin and Adamopoulos, 2021). However, even with modern diagnostic methods, the diagnostic sensitivity and specificity for axial spondyloarthritis are not higher than approximately 80% (Sieper and Poddubnyy, 2017). This means that a significant number of patients are still excluded from the current diagnostic criteria, and there is still a lot of room for improvement in our diagnostic methods. More importantly, it has been reported that approximately 10–20% of patients with non-radiographic axial spondyloarthritis will progress to AS within 1 year after the initial diagnosis while 20.3% of them will do so in 2–6 years (Sieper and Poddubnyy, 2017). Therefore, it is necessary to identify pre-AS patients, for identifying them could save more time for clinical interventions.

At present, our measures to identify axial spondyloarthritis are still limited beyond clinical features. Imaging (radiography, CT, and MRI), HLA-B27, and C-reactive protein (CRP) features are the main indices for the clinical diagnosis of axial spondyloarthritis (Zochling et al., 2005; Sieper and Poddubnyy, 2017; Ritchlin and Adamopoulos, 2021). More methods with high sensitivity and specificity are eagerly expected. Although with the rapid development of genomics technology, many serum biomarkers for the diagnosis of AS such as miR-214 (Kook et al., 2019), deoxyribonuclease 1-like 3 (Sun et al., 2020), anti-SIRT1 autoantibody (Hu et al., 2018), sclerostin (Perrotta et al., 2018), endoplasmic reticulum aminopeptidase 1 (Danve and O’Dell, 2015), and others have been identified, there is still a paucity of reliable indices for clinical practice besides HLA-B27 and CRP (Sieper and Poddubnyy, 2017; Danve and O’Dell, 2015). Therefore, the exploration of gene biomarkers of AS in peripheral blood is not only of real need and great practical value but could also deepen our knowledge of the pathophysiology of AS and even help us understand its etiology.

Thereby, in this study, we aimed to screen potential gene biomarkers in the peripheral blood by machine learning algorithms and build a diagnostic model and also preliminarily explore the immune microenvironment of AS to find some differences in immune cell proportions and potential explanations for our hub genes. To date, this work has not been done and reported; thus, it is imperative to bridge the knowledge gap in this area.

## Materials and methods

### Data collection

We searched the Gene Expression Omnibus (GEO) database (https://www.ncbi.nlm.nih.gov/geo/) for data sets containing whole-blood RNA expression data of normal people and AS patients with at least 15 samples in each group. Only GSE73754, GSE25101, and GSE18781 were qualified, and their expression and phenotype data were downloaded for subsequent studies. GSE73754 and GSE18781 contained whole-blood RNA expression data of 20 normal and 52 AS patients and 25 normal and 18 AS patients, respectively, together with their corresponding basic information such as sex and age. The expression data of GSE73754 were detected by the Illumina HumanHT-12 V4.0 expression BeadChip, University of Toronto, Canada, submitted on 06 Oct 2015. The expression data of GSE18781 were detected by the Affymetrix Human Genome U133 Plus 2.0 Array, Oregon Health & Science University, United States, submitted on 28 Oct 2009. GSE25101 contained whole-blood RNA expression data of 16 normal and 16 AS patients, which were detected by the Illumina HumanHT-12 V3.0 expression BeadChip, University of Queensland Diamantina Institute, Australia, submitted on 03 Nov 2010. However, the basic information of the subjects from GSE25101 was unavailable; so, it is only used as one of the validation sets. GSE11886 referred to the RNA expression data of *in vitro* cultured macrophages, which were obtained from the peripheral blood of nine normal people and eight AS patients. They were detected by the Affymetrix Human Genome U133 Plus 2.0 Array, Cincinnati Children’s Hospital Medical Center, United States, submitted on 25 Jun 2008. Although the RNA expression data of each set were normalized data, while in the quality control process, we found that samples of GSE18781 came from two batches; so, we used the “removebatcheffect” function of the “limma” package to recalculate the expression data (Ritchie et al., 2015).

### Identify common differentially expressed genes

Differentially expressed genes (DEGs) in GSE73754 and GSE25101 between normal people and AS patients were identified by the “limma” package (Ritchie et al., 2015) (cutoff value: the absolute value of log_2_foldchange >0.3 and *p*-value < 0.05). Then, common DEGs in GSE73754 and GSE25101 were selected as candidates for subsequent screening.

### Screening genes for diagnostic model by machine learning algorithms

GSE73754 served as the training set. Common DEGs were first screened by univariate logistic regression in the training set. Genes with a *p*-value < 0.05 were retained. Then, three machine learning algorithms: the least absolute shrinkage and selection operator (LASSO) logistic regression (Simon et al., 2011), a support vector machine recursive feature elimination (SVM-RFE) (Sanz et al., 2018), and random forest (RF) (Strobl et al. 2007) were adopted to screen hub genes. The common hub genes were selected as the final genes for the diagnostic model.

### Establishment of diagnostic model and its evaluation in training set and related validation set

A diagnostic model was established by the common hub genes and visualized by nomograms. Then, the prediction accuracy and discriminatory capacity were first assessed in GSE73754 and GSE25101 by the C-index, calibration analysis, receiver operating characteristic (ROC) curves, and decision curve analysis (DCA).

### Validation of model in validation sets

GSE18781 was set as an *in vivo* external validation set, while GSE11886 was set as an *in vitro* external validation set. The prediction accuracy and discriminatory capacity of the model were also assessed in the two aforementioned sets by the C-index, calibration analysis, ROC analysis, and DCA.

### Functional analysis of differentially expressed genes between normal and ankylosing spondylitis groups

GO and KEGG clustering and gene set enrichment analyses (GSEA) were used to explore the potential functions of the DEGs, which might indicate the causes of the difference between normal people and AS patients. With the same consideration, the protein–protein interaction (PPI) network analysis was also adopted to investigate the interaction between the proteins encoded by the DEGs (interaction score ≥0.4).

### Immune microenvironment analysis

The “CIBERSORT” package was employed to investigate the immune microenvironment (IME) of the samples. Meanwhile, the correlations between the different types of immune cells and the hub genes were also explored.

### Statistical analyses

In this study, the R software v3.63 was used to process data and generate charts. PPI network analyses were explored on the STRING website (https://cn.string-db.org/) (interaction score ≥0.4) and visualized by the Cytoscape software v3.7.1. Flexible statistical methods were adopted for the statistical analyses.

## Results

### Clinical characteristics of enrolled ankylosing spondylitis patients

The basic information of the samples from GSE73754 and GSE18781 is shown in Table 1. The clinical characteristics such as age and sex of the two sets were similar (*p*-value < 0.05).

**TABLE 1:** Clinical characteristics in training and validation sets.

Characteristics	Level	GSE18781	GSE73754	*p-*value	Test
Sample size (n)		43	72		
Sex	Female	25 (58.1)	35 (48.6)	0.342	Fisher test
	Male	18 (41.9)	37 (51.4)		
Age, median (interquartile range)		45.0 [32.5, 58.5]	41.5 [28.8, 51.2]	0.324	Kruskal test
Group	Normal	25 (58.1)	20 (27.8)		
	AS	18 (41.9)	52 (72.2)		

### Identification of hub genes

**FIGURE 1:**
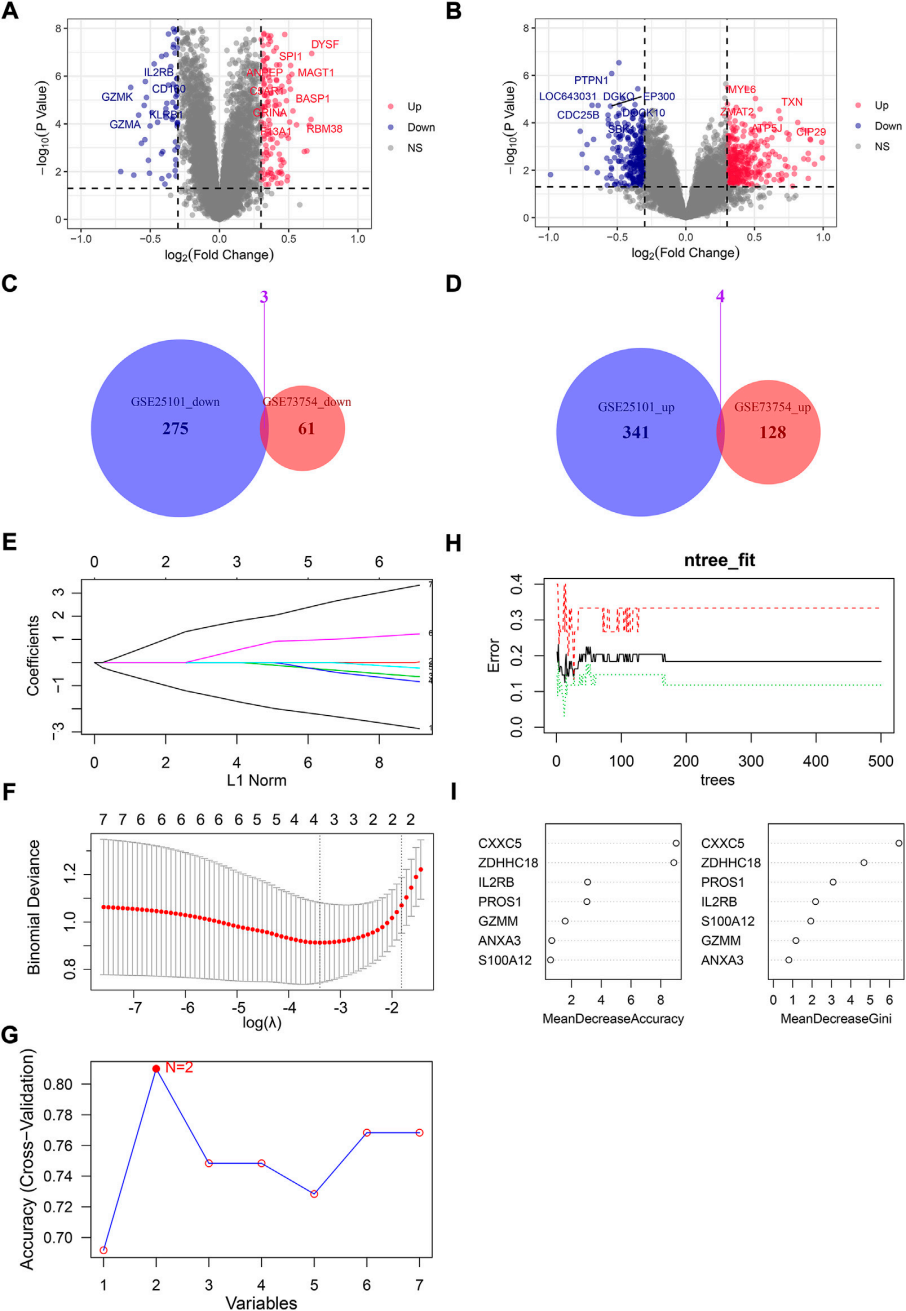
Screening for hub genes from DEGs between normal people and AS patients. The volcano plot for DEGs in GSE73754 **(A)** and GSE25101 **(B)**: *x*-axis represents log_2_ (fold change) of gene expressions in AS patients compared with normal controls, while the *y*-axis represents −log_10_ (*p*-value) of gene expression between AS patients and normal controls. **(C)** Venn plot for downregulated DEGs in GSE73754 and GSE25101. **(D)** Venn plot for upregulated DEGs in GSE73754 and GSE25101. **(E)** LASSO coefficient profiles for the seven common DEGs in the ten-fold cross-validations. **(F)** Partial likelihood deviance with changing of log(*λ*) plotted by LASSO regression in ten-fold cross-validations. **(G)** Filtering characteristic genes using the SVM-RFE algorithm: accuracy for models with different numbers of variables: the *x*-axis represents the number of variables involved in the models and the *y*-axis represents the corresponding accuracy of cross-validation of the models. **(H)** Relationship between the number of decision trees and the error rate of the model in RF. **(I)** Selecting hub genes by variable importance measures for RF.

In total, 64 downregulated and 132 upregulated DEGs were identified by “limma” in GSE73754 (Figure 1A). Also, 278 downregulated and 345 upregulated DEGs were identified in GSE25101 (Figure 1B). Then, the common upregulated and downregulated genes were selected: three common downregulated genes, namely, *IL2RB*, *GZMM*, and *CXXC5* (Figure 1C), and four common upregulated genes, namely, *S100A12*, *ANXA3*, *PROS1*, and *ZDHHC18* (Figure 1D).

Taking GSE73754 as the training set, the *p*-values of the seven genes in the univariate logistic regression were all lower than 0.05, meaning that all seven genes were qualified for the next screening. Then, they were screened by three different machine learning algorithms. *IL2RB*, *GZMM*, *S100A12*, and *ZDHHC18* were screened as hub genes by LASSO (*λ* = lambda.min) (Figures 1E,F). *IL2RB* and *ZDHHC18* were screened as hub genes by SVM-RFE (Figure 1G). *ZDHHC18*, *CXXC5*, *PROS1*, and *IL2RB* were screened as hub genes by RF with MeanDecreaseAccuracy >3 and MeanDecreaseGini >2 (mtry = 3, ntree = 200) (Figures 1H,I). Obviously, *IL2RB* and *ZDHHC18* were the common hub genes screened by the three algorithms, and they were selected as the final hub genes for a diagnostic model in AS.

### Evaluation of diagnostic model in training set (GSE73754) and GSE25101

**FIGURE 2:**
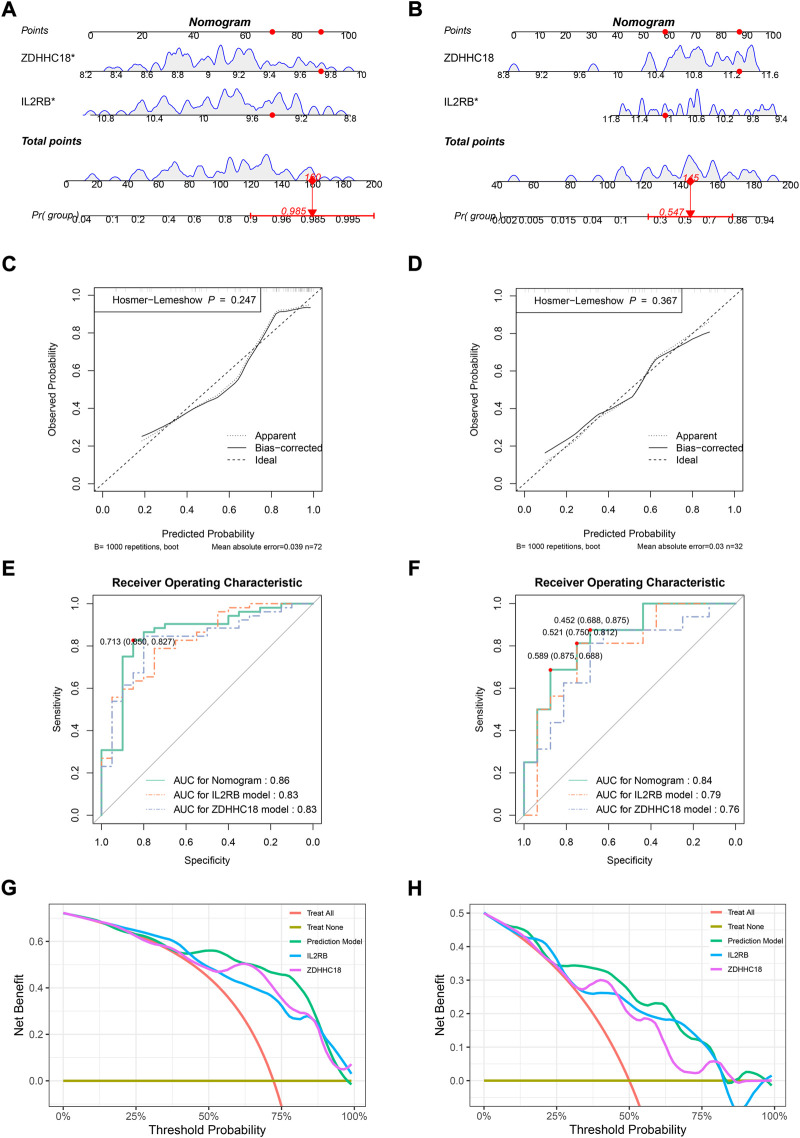
Evaluating the diagnostic model in the training set and a related validation set. Nomograms for the diagnostic model in GSE73754 **(A)** and GSE25101 **(B)**. Calibration plots for the diagnostic model in GSE73754 **(C)** and GSE25101 **(D)**: *x*-axis represents the predicted probability of AS by the model, while the *y*-axis represents the observed probability of AS, the diagonal (dashed line) represents the ideal status that the predicted probability equaled the observed probability, and the solid and dotted lines represent the apparent and bias-corrected statuses of the predicted and observed probabilities, respectively. ROC plots for the diagnostic model in GSE73754 **(E)** and GSE25101 **(F)**: the *x*-axis represents 1-specificity of the model, while the *y*-axis represents the sensitivity of the model. DCA in GSE73754 **(G)** and GSE25101 **(H)**: the *x*-axis represents the threshold probability for the treatment or intervention, while the *y*-axis represents the net benefit.

A diagnostic model was established by *IL2RB* and *ZDHHC18* and then visualized by a nomogram in GSE73754 (Figure 2A) and GSE25101 (Figure 2B), respectively. The nomograms suggested that the higher the expression level of *ZDHHC18* was, the higher was the risk of AS, while the reverse was true for *IL2RB*. The C-index of the diagnostic model in GSE73754 was 0.86 (95% CI: 0.76–0.96) and 0.84 (95% CI: 0.71–0.97) in GSE25101. The calibration analyses showed that the predicted probability was in high agreement with the observed probability, suggesting a high accuracy of the model both in the training set and an external validation set (Figures 2C,D).

The ROC analysis in GSE73754 showed that the areas under the curves (AUCs) for the nomogram, *IL2RB*, and *ZDHHC18* were 0.86, 0.83, and 0.83, respectively (Figure 2E). The optimal truncation value of Y was 0.713, and the corresponding specificity and sensitivity were 0.85 and 0.827, respectively (formula: y = 2.9111*EXP_*ZDHHC18*_ − 2.3256*EXP_*IL2RB*_ − 2.2376, where EXP_*ZDHHC18*_ refers to the expression value of *ZDHHC18* and EXP_*IL2RB*_ refers to the expression value of *IL2RB*). In this model, the value of Y ≥ 0.713, predicted to be AS, was otherwise normal. The actual prediction accuracy of the model in GSE73754 was 0.82. While in GSE25101, the AUCs for the nomogram, *IL2RB*, and *ZDHHC18* were 0.84, 0.79, and 0.76, respectively (formula: y = 2.320,052*EXP_*ZDHHC18*_ − 1.728,388*EXP_*IL2RB*_ − 6.902,309) (Figure 2F). There were three optimal truncation values for Y: 0.589 with a corresponding specificity of 0.875 and sensitivity of 0.688, 0.521 with a corresponding specificity of 0.75 and sensitivity of 0.812, and 0.452 with a corresponding specificity of 0.688 and sensitivity of 0.875. The actual prediction accuracy of the model in GSE25101 was 0.72. The DCA for the nomogram and models involved only one of these genes, which indicated that the net benefit of the nomogram was higher than that of the other models (Figures 2G,H).

### Validation of model in independent cohort and *in vitro*

**FIGURE 3:**
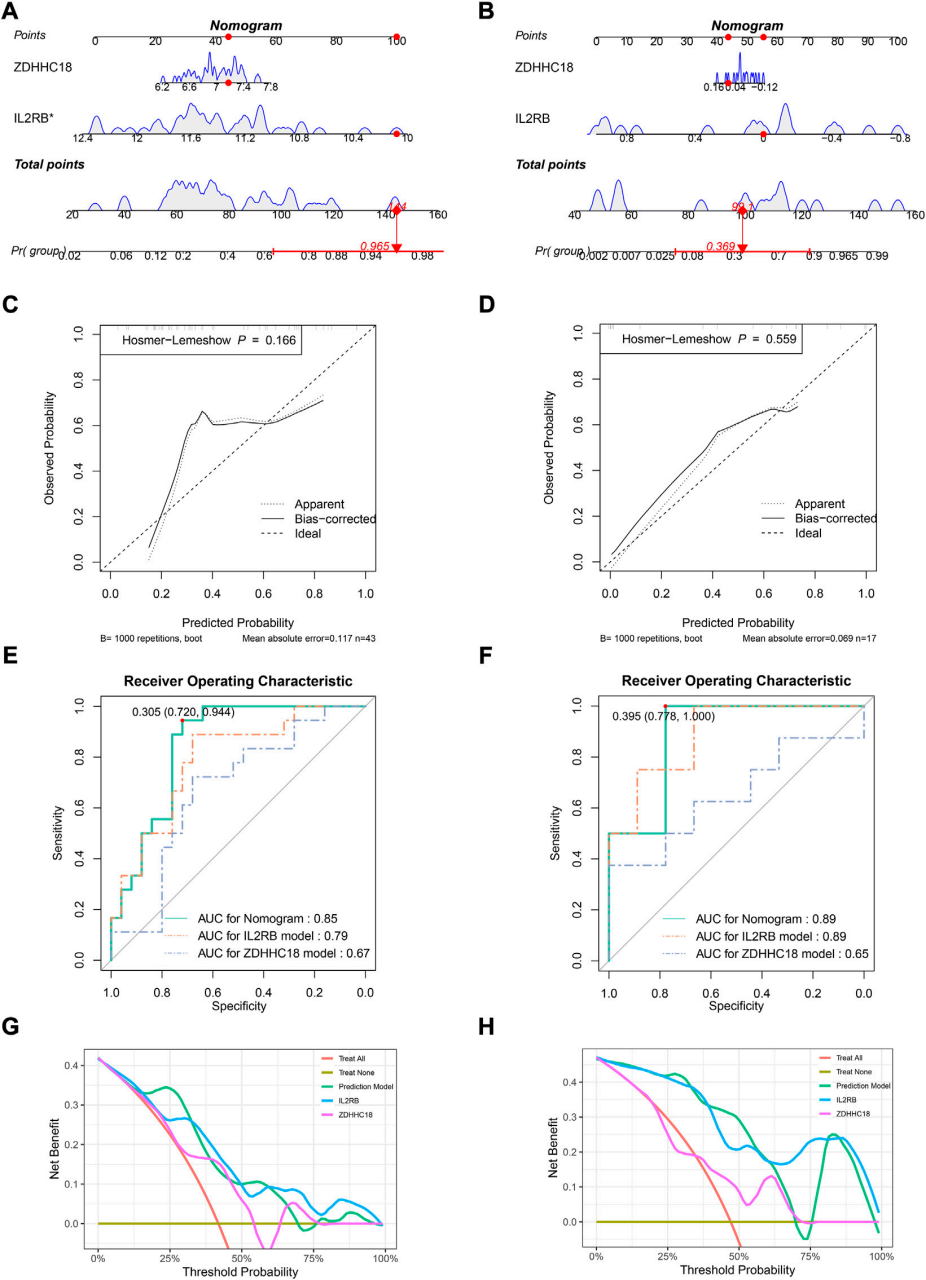
Validating the diagnostic model in validation sets. Nomograms for the diagnostic model in GSE18781 **(A)** and GSE11886 **(B)**. Calibration plots for the diagnostic model in GSE18781 **(C)** and GSE11886 **(D)**: the *x*-axis represents the predicted probability of AS by the model, while the *y*-axis represents the observed probability of AS. The diagonal (dashed line) represents the ideal status that the predicted probability equaled the observed probability, and the solid and dotted lines represent the apparent and bias-corrected statuses of the predicted and observed probabilities, respectively. ROC plots for the diagnostic model in GSE18781 **(E)** and GSE11886 **(F)**: the *x*-axis represents 1-specificity of the model, while the *y*-axis represents the sensitivity of the model. DCA in GSE18781 **(G)** and GSE11886 **(H)**: the *x*-axis represents the threshold probability of the treatment or intervention, while the *y*-axis represents the net benefit.

The model was validated in an independent cohort, GSE18781, and *in vitro* cohort, GSE11886. The nomogram for GSE18781 supported the conclusion reached in the training set that AS patients had a higher expression of *ZDHHC18* and lower expression of *IL2RB* (Figure 3A). The function of *IL2RB* in GSE11886 was in accordance with that in the other sets; however, the function of *ZDHHC18 in vitro* was opposite to that *in vivo*, and this might have been due to the lack of the *in vivo* microenvironment (Figure 3B). According to the coverage of points in the nomogram, *IL2RB* showed higher weight in the validation sets and the alteration between the nomograms also indicated that it is a more robust indicator than *ZDHHC18*. The C-index of the diagnostic model in GSE18781 was 0.85 (95% CI: 0.73–0.96) and 0.89 (95% CI: 0.73–1.05) in GSE11886. The calibration analyses revealed that the prediction accuracy of the model was lower than that in GSE73754 and GSE25101; however, it still had acceptable accuracy (Figures 3C,D).

The ROC analysis in GSE18781 revealed that the areas under the curves (AUCs) for the nomogram, *IL2RB*, and *ZDHHC18* were 0.85, 0.79, and 0.67, respectively (Figure 3E). The optimal truncation value of Y was 0.305, and the corresponding specificity and sensitivity were 0.72 and 0.994, respectively (formula: y = 1.29499*EXP_*ZDHHC18*_ − 2.582,298*EXP_*IL2RB*_ + 20.055204). The actual prediction accuracy of the model in GSE18781 was 0.72. While in GSE11886, the AUCs for nomogram, *IL2RB*, and *ZDHHC18* were 0.89, 0.89, and 0.65, respectively (formula: y = −6.49159*EXP_*ZDHHC18*_ − 6.13506*EXP_*IL2RB*_ − 0.01334) (Figure 3F). The optimal truncation value of Y was 0.395, and the corresponding specificity and sensitivity were 0.778 and 1, respectively. The actual prediction accuracy of the model in GSE11886 was 0.76. The DCA showed that patients could get a high net benefit from the nomogram (Figures 3G,H). Besides, a high net benefit could also be obtained from the model established by IL2RB only in this set.

### Results of functional analysis of differentially expressed genes between normal and ankylosing spondylitis groups

**FIGURE 4:**
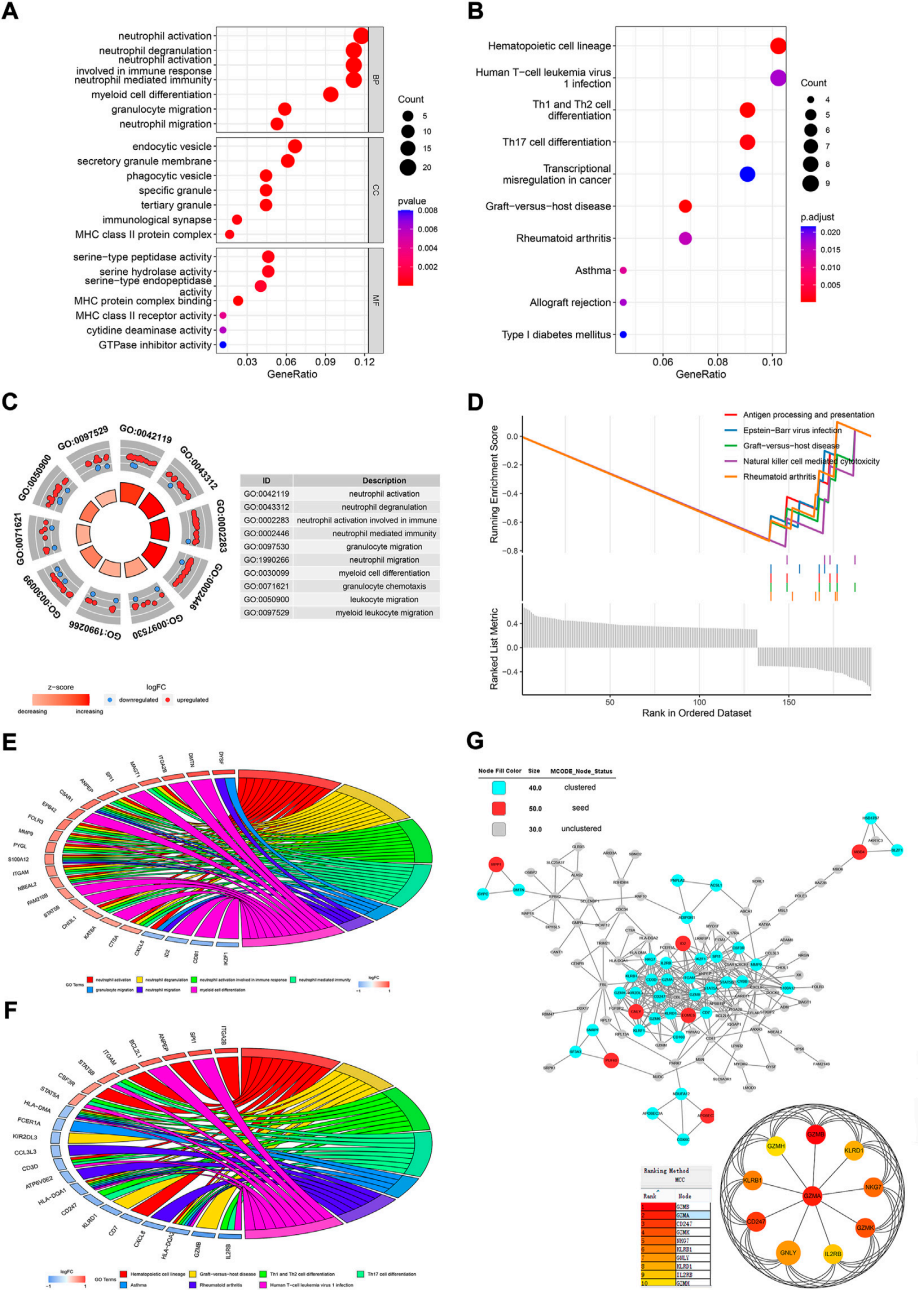
Functional analysis of the DEGs between normal people and AS patients. Dot plots for GO **(A)** and KEGG **(B)** analyses of DEGs. **(C)** Circle plot for BP clustering of the DEGs. **(D)** GSEA analysis for the DEGs. **(E)** Chord plot for the top seven clustered GO terms. **(F)** Chord plot for the top seven clustered KEGG pathways. **(G)** PPI network analysis for DEGs.

There was a total of 196 DEGs between normal people and AS patients in GSE73754. Biological process (BP) clustering of the DEGs showed that they were mainly clustered in neutrophil activation, degranulation, immune response, and migration (Figure 4A). Myeloid cell differentiation, leukocyte migration, and granulocyte migration were also clustered BPs. Gene clustering of cellular components (CC) was mostly in the area of membranes, such as endocytic vesicles, secretory granule membranes, membrane microdomains, and cytoplasmic vesicle lumens (Figure 4A). Molecular functions (MFs) of the DEGs were mostly clustered in serine-type peptidase activity, serine hydrolase activity, serine-type endopeptidase activity, and MHC protein complex binding (Figure 4A). In the KEGG clustering of the DEGs, the hematopoietic cell lineage, human T-cell leukemia virus 1 infection, Th1 and Th2 cell differentiation, and Th17 cell differentiation were the top clustered pathways (Figure 4B). The circle plot for BP clustering showed that neutrophil activation, degranulation, immune response, and migration were upregulated in AS (Figure 4C). By GSEA, antigen processing and presentation, natural killer cell–mediated cytotoxicity, graft-*versus*-host disease, Epstein–Barr virus infection, and rheumatoid arthritis were the top enriched gene sets, which were all downregulated in AS patients (Figure 4D). The top three upregulated pathways enriched with core enrichment genes were neutrophil extracellular trap formation, complement and coagulation cascades, and the rap1 signaling pathway. The GO chord plot showed that *DYSF*, *DMTN*, *ITGA2B*, *MAGT1*, *SPI1*, *CXCL8*, *ID2*, *CD81*, *IKZF1*, and many others were involved in the top seven GO terms (Figure 4E). The KEGG chord plot showed that *ITGA2B*, *SPI1*, *ANPEP*, *BCL2L1*, *STAT5B*, *IL2RB*, *GZMB*, *HLA-DQA2*, *CXCL8*, and many more were involved in the top seven KEGG terms (Figure 4F).

The PPI network of the proteins encoded by DEGs showed that MMP1, ID2, MBD4, GNLY, EOMES, PUF60, and APOBEC3G were seed proteins in the network by the MCODE application in Cytoscape (Figure 4G). The cyan nodes were also pivotal nodes in the net, such as IL2RB, GZMA, SPI1, and many others. Then, GZMA, IL2RB, CD247, KLRB1, GZMH, GZMB, GZMK, KLRD1, NKG7, and GNLY were the top 10 hub proteins screened by cytoHubba.

### Results of immune microenvironment analyses

**FIGURE 5:**
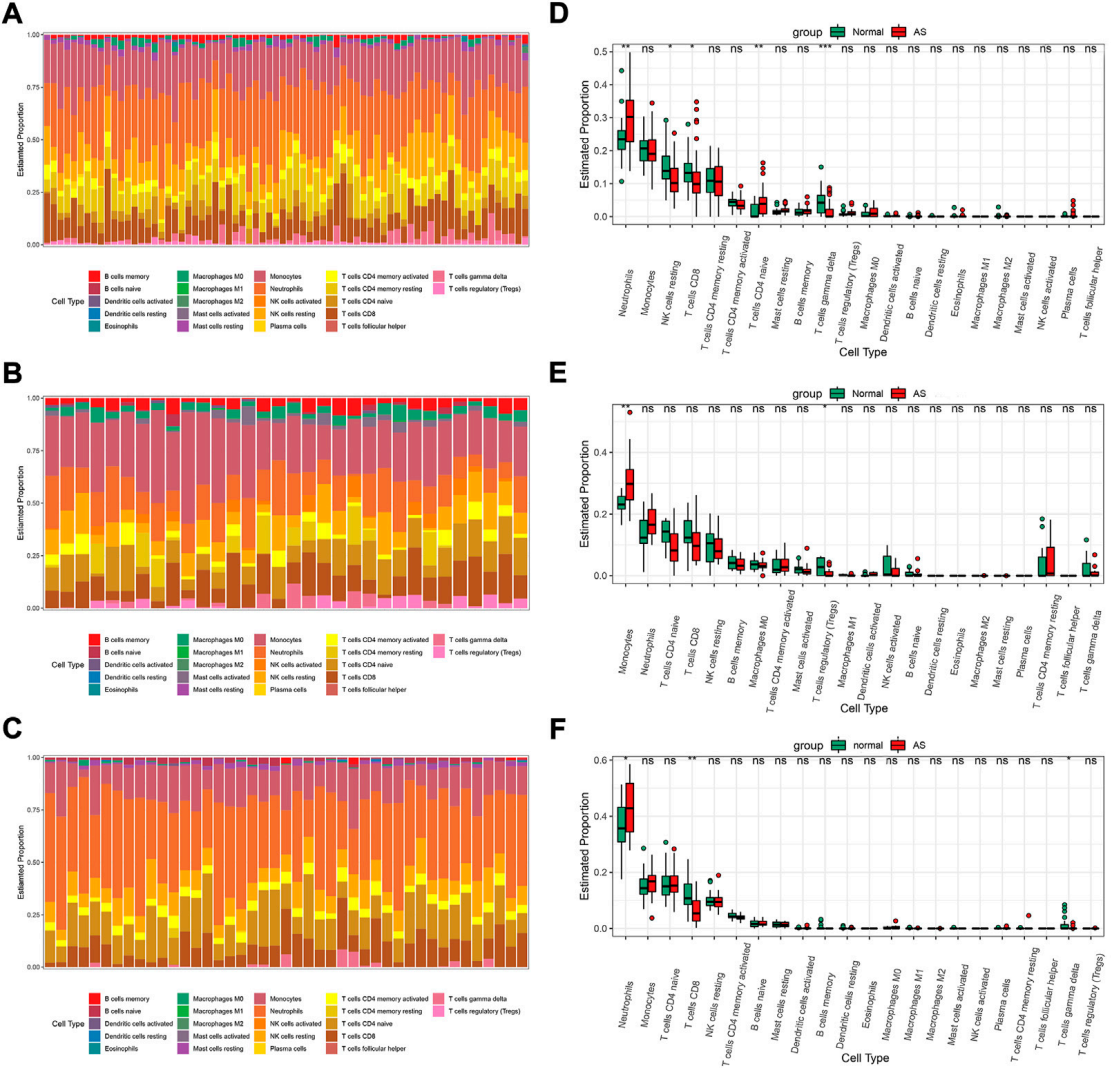
The IME analysis of the sets by CIBERSORT. The proportion of the 22 immune cells for samples in GSE73754 **(A)**, GSE25101 **(B)**, and GSE18781 **(C)**. Boxplots for the 22 immune cells between normal people and AS patients in GSE73754 **(D)**, GSE25101 **(E)**, and GSE18781 **(F)** (p significance level: no significance (ns), *p* ≥ 0.05; *, *p* < 0.05; **, *p* < 0.01; ***, *p* < 0.001; ****, *p* < 0.0001.).

IME analyses were performed in GSE73754, GSE25101, and GSE18781 by CIBERSORT. The proportions of the 22 immune cells for samples are shown in Figures 5A–C. In all three sets, the neutrophils and monocytes accounted for the top two highest proportions and together made up the majority of the immune cells, while the other immune cells such as granulocytes, B cells, dendritic cells, and macrophages each made up only a small proportion of the total immune cell population. The relative quantities of different immune cells in normal people and AS patients are shown in Figures 5D–F. In GSE73754, when compared with the normal subjects, there were more neutrophils and naive CD4 T cells detected in the blood of AS patients, while there were fewer resting NK, CD8^+^ T, and gamma-delta T cells (Figure 5D). In GSE25101, monocytes were found to be more in the blood of AS patients, while regulatory T cells (Tregs) were fewer. In this set, the relative number of neutrophils was more in the AS group; however, the difference was not statistically significant (Figure 5E). The result in GSE18781 was similar to that in GSE73754; the relative number of neutrophils was increased, while that of CD8^+^ and gamma-delta T cells was decreased in patients with AS (Figure 5F).

**FIGURE 6:**
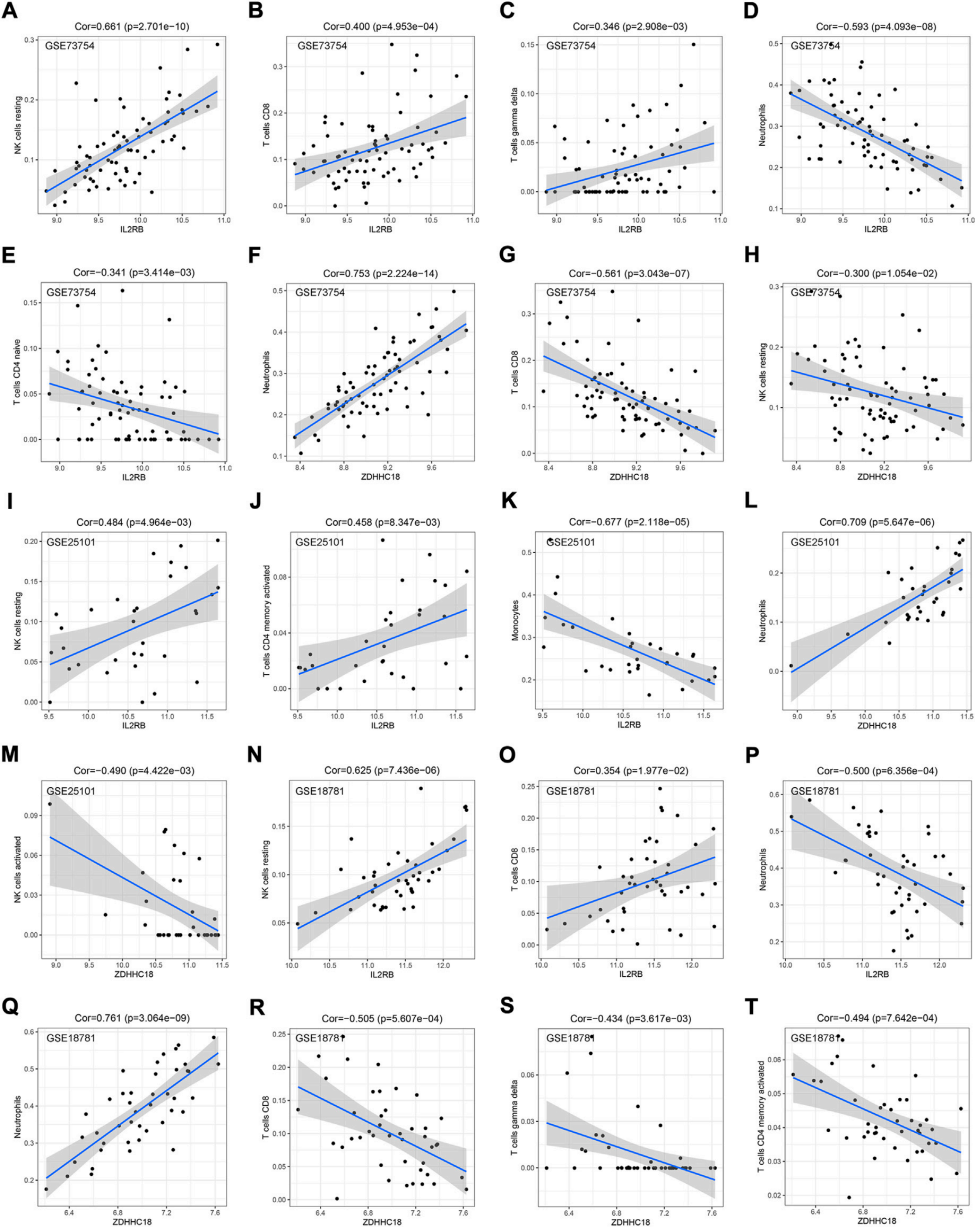
Correlation between hub genes and IME cells. The correlation between the expression of IL2RB and the estimated proportion of resting NK cells **(A)**, CD8^+^ T cells **(B)**, gamma-delta T cells **(C)**, neutrophils **(D)**, and native CD4 T cells **(E)** by CIBERSORT in GSE73754. The correlation between the expression of ZDHHC18 and the estimated proportions of neutrophils **(F)**, CD8^+^ T cells **(G)**, and resting NK cells **(H)** by CIBERSORT in GSE73754. The correlation between the expression of IL2RB and the estimated proportions of resting NK cells **(I)**, activated CD4^+^ memory T cells **(J)**, and monocytes **(K)** by CIBERSORT in GSE25101. The correlation between the expression of ZDHHC18 and the estimated proportions of neutrophils **(L)** and activated NK cells **(M)** by CIBERSORT in GSE25101. The correlation between the expression of IL2RB and the estimated proportions of resting NK cells **(N)**, CD8^+^ T cells **(O)**, and neutrophils **(P)** by CIBERSORT in GSE18781. The correlation between the expression of ZDHHC18 and the estimated proportions of neutrophils **(Q)**, CD8^+^ T cells **(R)**, gamma-delta T cells **(S)**, and activated CD4^+^ memory T cells **(T)** by CIBERSORT in GSE18781.

The correlation between our hub genes (*IL2RB* and *ZDHHC18*) and immune cells was also explored. In GSE73754, the expression of *IL2RB* was positively correlated with the relative numbers of resting NK, CD8^+^ T, and gamma-delta cells (Figures 6A–C) and negatively correlated with the relative numbers of neutrophils, naive CD4 T cells, and monocytes (Figures 6D,E). Meanwhile, the expression of *ZDHHC18* was positively correlated with the relative number of neutrophils (Figure 6F) but negatively correlated with the relative numbers of CD8^+^ T cells and resting NK cells (Figures 6G,H). In GSE25101, the expression of *IL2RB* was positively correlated with the relative numbers of resting NK and activated CD4^+^ memory T cells (Figures 6I,J) and negatively correlated with the relative number of monocytes (Figure 6K). Besides, the expression of *ZDHHC18* was positively correlated with the relative number of neutrophils (Figure 6L) but negatively correlated with the relative number of activated NK cells (Figure 6M). There was no significant correlation between Tregs and the hub genes. Lastly, in GSE18781, the expression of *IL2RB* was positively correlated with the relative numbers of resting NK and CD8^+^ T cells (Figures 6N,O) and negatively correlated with the relative number of neutrophils (Figure 6P). Moreover, the expression of *ZDHHC18* was positively correlated with the relative number of neutrophils (Figure 6Q) but negatively correlated with the relative quantities of CD8^+^, gamma-delta, and activated CD4^+^ memory T cells (Figures 6R–T).

## Discussion

It is known that AS is an inflammatory disease mainly involving the axial skeleton’s joints and entheses. The essential change in AS is the dysregulation of inflammation by innate and adaptive immune responses (Mauro et al., 2021). Although AS is primarily associated with the axial skeleton, recent research indicates that it may be initiated in the gut (Yang et al., 2016a). Besides, the peripheral and extra-articular manifestations of AS also suggest that it is a systemic disorder. Therefore, DEGs in the peripheral blood of AS patients can also reflect some features of AS. As for RNAs extracted from the peripheral blood, they are mostly from the nucleated cells in the blood, similar to leukocytes and immature red blood cells; so, it is rational to explore the immune microenvironment of the blood of AS patients. More importantly, compared with the focal tissue, the peripheral blood is easier to obtain and a more commonly used clinical detection material, which is also conducive to the transition from experimental results to applications.

To date, HLA-B27 is still considered the most important factor in the pathogenesis of AS (Colbert et al., 2010; Bowness, 2015; Pedersen and Maksymowych, 2019; Sharip and Kunz, 2020; Voruganti and Bowness, 2020). First, many shreds of evidence supported the hypothesis that the alternation of the amino acid sequence in the antigenic peptide-binding groove of HLA-B27 might induce changes in the binding specificity of peptides and result in CD8^+^ T cell–mediated immune cross-reactivity in the AS focus (Mear et al., 1999; Guiliano et al., 2017). Second, endoplasmic reticulum stress was induced by the accumulation of misfolded HLA-B27, which led to an unfolded protein response (UPR) and autophagy (Yu et al., 2017). Third, the HLA-B27 homodimer hypothesis suggests that the HLA-B27 homodimer could activate CD8^+^ T cells and NK cells by the specific receptors on their surfaces, activating the IL-23/IL-17 axis (Bowness et al., 2011). Certainly, there were also many other hypotheses, such as the non-MHC hypothesis. However, the point of intersection is that all the hypotheses are focused on the antigen-presenting process, and its failure or dysfunction would mostly result in the activation of the TNF signaling pathway and the IL23/IL17 axis and eventually lead to the AS phenotype. However, the sensitivity and specificity of HLA-B27 alone were relatively low.

Here, to enhance the reliability and stability of the results, only common genes screened by the three machine learning algorithms were selected as hub genes for a diagnostic model. The three methods used in our study are the most popular and widely used ones in bioinformatics analyses. Currently, deep learning methods are also popular in bioinformatics analyses, and some of them can even generate different methods based on machine learning techniques such as BioSeq-BLM and ilearn. However, they are limited by the quantity and quality of the training data and are more suitable for large data processing (Choi et al., 2020). The data used in our study are small; so, the three machine learning methods could be more suitable. Meanwhile, deep learning methods are more complex, time-consuming, have high requirements for computer hardware, and have results that are more difficult to interpret (Choi et al., 2020). Besides, we validated the model in three different data sets: one related data set, one independent data set, and one data set of *in vitro* samples to further assess the predictive reliability and stability of the model. The C-index, calibration analysis, ROC analysis, and DCA in the training and validation sets suggested that it is an excellent diagnostic model with good applicability.

Functional analyses of DEGs and IME analyses indicated that neutrophil activation, migration, and degranulation were activated in AS patients. Also, the relative number or proportion of neutrophils was significantly higher in AS patients. Our result is also confirmed by other researchers who have also suggested that the neutrophil-to-lymphocyte ratio be used as an indicator of AS activity (Mercan et al., 2016; Xu et al., 2020; Gökmen et al., 2015). Meanwhile, neutrophil extracellular trap formation and the complement and coagulation cascades were also upregulated in AS, which might induce an autoimmune response, and this is in agreement with the IME analysis result and our current understanding of AS (Gonnet-Gracia et al., 2008; Yang et al., 2016b). A potential explanation for the aforementioned finding is that the increased number of neutrophils might release excessive IL-17A, the key cytokine in the pathogenesis of AS. Although mature neutrophils lack the transcriptional machinery to produce IL-17A, they could produce and store IL-17A before they mature and accumulate it from the extracellular environment (Lin et al., 2011; Tamassia et al., 2018). Besides, in GSE25101, monocytes were also found to be more numerous in AS patients with DEGs clustered in myeloid cell differentiation and leukocyte migration in GO clustering. It is known that monocytes share some similar functions with neutrophils in immune response, and there have also been reports that the monocyte-to-lymphocyte ratio was increased in AS patients (Huang et al., 2018; Wang et al., 2021; Liang et al., 2021). Whether or not the increments in the number of lymphocytes and monocytes are two different subtypes of AS remains unknown.

**FIGURE 7:**
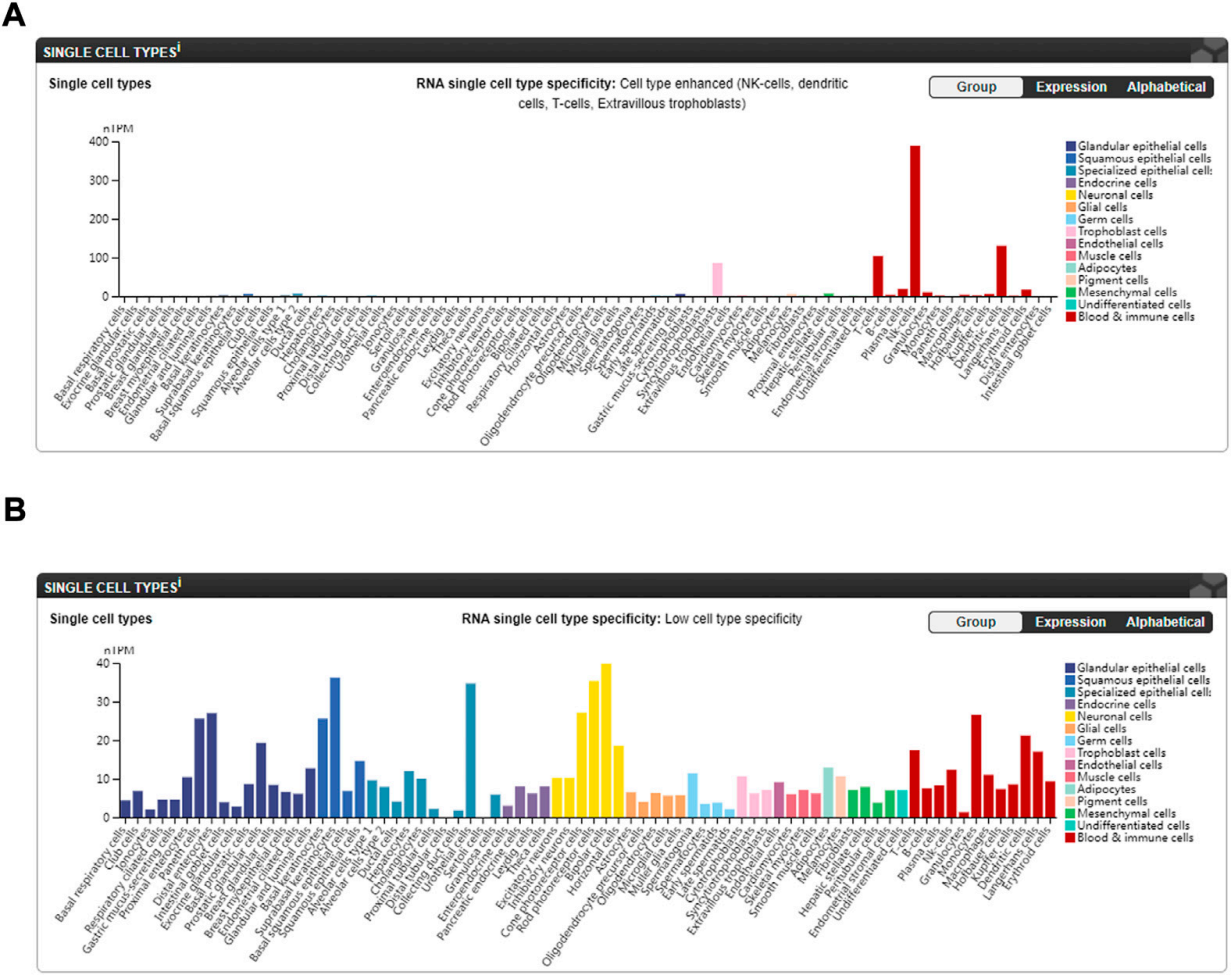
Expression of hub genes in different single-cell types of normal subjects from the HPA website (Karlsson et al., 2021). **(A)** Expression of IL2RB in different single-cell types (available from v21.1. proteinatlas.org: https://www.proteinatlas.org/ENSG00000100385-IL2RB/single+cell+type). **(B)** Expression of ZDHHC18 in different single-cell types (available from v21.1. proteinatlas.org: https://www.proteinatlas.org/ENSG00000204160-ZDHHC18/single+cell+type).

Lastly, *IL2RB* is a hub gene both in GO/KEGG clustering and the PPI network analysis. Its expression was positively correlated with the relative quantities/proportions of resting NK cells and negatively correlated with the relative quantities/proportions of neutrophils and monocytes in our study, which is in line with the data from the Human Protein Atlas (HPA) website (Karlsson et al., 2021) (Figure 7A: available from v21.1.proteinatlas.org, https://www.proteinatlas.org/ENSG00000100385-IL2RB/single+cell+type). While *ZDHHC18* was observed to be positively correlated with the relative quantities/proportions of neutrophils in all three sets, it did not seem to be highly expressed in granulocytes based on the data from the HPA website (Karlsson et al., 2021) (Figure 7B: available from v21.1.proteinatlas.org, https://www.proteinatlas.org/ENSG00000100385-IL2RB/single+cell+type). Above all, our results suggests that *IL2RB* might be correlated with AS *via* the suppression of the function of resting NK cells, and *ZDHHC18* might be correlated with AS through the function of neutrophils; however, the detailed underlying mechanism still needs to be studied further.

In this study, *IL2RB* and *ZDHHC18* were the two finally screened hub genes. The former had already been identified by other researchers as one of the hub genes in AS (Zhu et al., 2013; Zheng et al., 2021), while to the best of our knowledge, the latter was first reported here by us. *IL2RB*, interleukin 2 receptor subunit beta, encoded the beta subunit of a heterodimer or heterotrimer receptor involved in T cell–mediated immune responses and is probably involved in the stimulation of neutrophil phagocytosis by *IL15* (Ratthé and Girard, 2004; Zhang et al., 2019). This protein is a type-I membrane protein primarily expressed in NK cells, T cells, and dendritic cells. According to the KEGG database (https://www.kegg.jp/), *IL2RB* was involved in many pathways, which include endocytosis, the PI3K-Akt signaling pathway, the JAK-STAT signaling pathway, Th1 and Th2 cell differentiation, Th17 cell differentiation, and many more. Obviously, Th1 and Th2 cell differentiation and Th17 cell differentiation seemed to be most related to AS, such that IL2 signaling can inhibit the differentiation of Th17 *via* the inhibition of the transcription factor *RORγt* (Waldmann, 2006; Soper et al., 2007; Liao et al., 2011; Allard-Chamard et al., 2020; Pol et al., 2020). Therefore, with the downregulation of *IL2RB* in this study, Th17 was anticipated to be expanded. However, Th1 and Th2 cell differentiation and Th17 cell differentiation were observed to be downregulated in GSEA (Figure 6D), which is contradictory to our knowledge of AS; therefore, something should be noticed. On the one hand, the pathogeneses of changes in AS are mainly involved in the focus of AS, not in the circulatory system, and our knowledge was largely based on that; so, it might be common for samples from the two sites to have some differences. On the other hand, the role of *IL2* signaling in the differentiation of Th17 has still not been fully clarified (Campbell and Bryceson, 2019). The question is what was the minimum *IL2* signal required to maintain the Treg numbers. Isabel Z Fernandez et al. reported a hypomorphic mutation of *IL2RB* in two infant siblings that resulted in an anticipated reduction in Tregs and an expansion of immature NK cells (Fernandez et al., 2019). Here, in two of the three sets, the relative numbers of CD8^+^ and gamma-delta T cells were decreased, while that of Tregs was not significantly reduced, which might indicate that the reduced *IL2* signal was still adequate for the proliferation of Tregs and the suppression of effector T-cell expansion (Figures 5D,F). Besides, *via* the blockade of *IL-2 in vitro* and *in vivo*, Kenjiro Fujimura et al. found that the number of Th17 cells did not significantly increase but the proportion of Th17 cells did, which suggests that it might increase the proportion of Th17 by suppressing the total number of immune cells (Fujimura et al., 2013). In this study, the numbers of certain kinds of T cells, such as CD8^+^ T cells gamma-delta T cells, and Tregs, were observed to have decreased in AS patients, and this might overwhelm the effect of the downregulation of Th17 cell differentiation. However, to see which type of immune cells became fewer and if this would affect the synthesis of IL17 by Th17 cells in AS patients requires further research. In the end, although the potential function of *IL2RB* in AS remains unclear, it might contribute to AS by reducing the number of Treg cells and relatively increasing the proportion of Th17 cells, thereby activating the *IL17* signaling to form AS phenotypes.

*ZDHHC18*, zinc finger DHHC-type palmitoyltransferase 18, encoded a palmitoyltransferase, which was involved in peptidyl-l-cysteine S-palmitoylation (Ohno et al., 2012). Studies on *ZDHHC18* are rare and insufficient. Currently, it is reported to be associated with innate immunity (Shi et al., 2022), glioma (Chen et al., 2019), ovarian cancer (Pei et al., 2022), and schizophrenia (Zhao et al., 2018). The common palmitoylation substrates of ZDHHC18 are HRAS and LCK (Baumgart et al., 2010; Akimzhanov and Boehning, 2015; Adachi et al., 2016). Palmitoylated HRAS could be translocated and stably anchored to the plasma membrane (Yang et al., 2020), while palmitoylation-defective HRAS was trapped in the Golgi apparatus and was unable to traverse to the plasma membrane. Meanwhile, *ZDHHC18* could activate the rap1 signaling pathway by the palmitoylation of Ras and promote the proliferation of cells, which was consistent with our GSEA result. Besides, Rac1, which was also involved in the rap1 signaling pathway mainly by regulating cell adhesion, migration, and polarity, could also be palmitoylated by the ZDHHC family (Yang et al., 2020). Though we currently do not know the exact role of *ZDHHC18* in AS, it is essential for neutrophil motility as well as directional sensing during migration, which was clustered by GO clustering in our study. In addition, the palmitoylation of LCK could promote T-cell receptor signaling to activate T cells, although this was not seen in our study, which meant that it is not important in the pathogenesis of AS. Furthermore, *ZDHHC18* could negatively regulate CGAS-STING signaling–mediated antiviral innate immunity *via* the palmitoylation of cGAS, which means that the antiviral immunity in AS patients might be impaired by the high expression of *ZDHHC18* (Shi et al., 2022). In our study, KEGG and GSEA also indicated dysregulation in some antiviral immune pathways.

In general, our study indicated that *IL2RB* might be involved in the pathogenesis of AS through the *IL2* signaling pathway and *ZDHHC18* through the rap1 signaling pathway. Both of these could be used as potential biomarkers in AS. Meanwhile, it should also be noted that although we explored some changes in RNA expression in the peripheral blood of AS patients, it is only just the tip of the iceberg. Therefore, more validations of the two genes in AS patients are required, and the mechanisms of these two genes in the pathogenesis of AS also require further research. These are the two main directions of our subsequent research.

## Conclusion

*IL2RB* and *ZDHHC18* were identified as potential blood biomarkers of AS, which might be used for the early diagnosis of AS and serve as supplements to the existing diagnostic methods. Our study helps deepen the understanding of the pathogenesis of AS.

## Data Availability

Publicly available data sets were analyzed in this study. These data can be found at: https://www.ncbi.nlm.nih.gov/geo/query/acc.cgi?acc=GSE73754 https://www.ncbi.nlm.nih.gov/geo/query/acc.cgi?acc=GSE25101 https://www.ncbi.nlm.nih.gov/geo/query/acc.cgi?acc=GSE18781 https://www.ncbi.nlm.nih.gov/geo/query/acc.cgi?acc=GSE11886.

## Abbreviations

AS, ankylosing spondylitis; AUC(s), area under curve(s); BP, biological process; CC, cellular component; CI, confidence interval; DCA, decision curve analysis; DEGs, differentially expressed genes; GEO, Gene Expression Omnibus database; GO, Gene ontology; GSEA, Gene Set Enrichment Analysis; HPA, Human Protein Atlas; KEGG, Kyoto Encyclopedia of Genes and Genomes; KM, Kaplan–Meier; LASSO, least absolute shrinkage and selection operator; MF, molecular function; OS, overall survival; PPI, protein–protein interaction; RF, random forest; ROC, receiver operating characteristic; Tregs, regulatory T cells.
